# Conversational presentation mode increases credibility judgements during information search with ChatGPT

**DOI:** 10.1038/s41598-024-67829-6

**Published:** 2024-07-25

**Authors:** Christine Anderl, Stefanie H. Klein, Büsra Sarigül, Frank M. Schneider, Junyi Han, Paul L. Fiedler, Sonja Utz

**Affiliations:** 1 Leibniz-Institut Für Wissensmedien (IWM), Schleichstraße 6, 72076 Tübingen, Germany; 2 Eberhard Karls Universität Tübingen, Tübingen, Germany; 3 Present Address: University of Amsterdam, Amsterdam, The Netherlands; 4 Present Address: Eberhard Karls Universität Tübingen, Tübingen, Germany

**Keywords:** Psychology, Human behaviour

## Abstract

People increasingly use large language model (LLM)-based conversational agents to obtain information. However, the information these models provide is not always factually accurate. Thus, it is critical to understand what helps users adequately assess the credibility of the provided information. Here, we report the results of two preregistered experiments in which participants rated the credibility of accurate versus partially inaccurate information ostensibly provided by a dynamic text-based LLM-powered agent, a voice-based agent, or a static text-based online encyclopedia. We found that people were better at detecting inaccuracies when identical information was provided as static text compared to both types of conversational agents, regardless of whether information search applications were branded (ChatGPT, Alexa, and Wikipedia) or unbranded. Mediation analysis overall corroborated the interpretation that a conversational nature poses a threat to adequate credibility judgments. Our research highlights the importance of presentation mode when dealing with misinformation.

## Introduction

When OpenAI released their large language model (LLM)-powered conversational agent ChatGPT in November 2022, it was adopted faster than any prior medium or technology— within two months, ChatGPT had 100 million active users^1^. Since then, LLMs have become ubiquitous in both public and academic discourse, and multiple companies have launched their own LLM-based conversational agents. Because LLMs interpret human language in a way that allows them to take into account user intent and the contextual meaning of terms, enabling unprecedentedly human-like conversations, they are increasingly used for information enquiries. Indeed, their potentially disruptive implications for search engines are widely discussed^2^. However, LLMs are known to “hallucinate” at times, that is, to make up information—often convincingly so^3–5^. Therefore, it is crucial to understand what helps accurately assess the credibility of the provided information.

We address this question in two preregistered experiments. While prior research descriptively explored the accuracy of responses provided by ChatGPT or the percentage of made-up information in narrowly defined areas^3,6,7^, the present work focuses on how the presentation mode of information influences credibility judgments. We build on prior theorizing and empirical work demonstrating that the modality in which information is presented affects credibility judgments^8,9^ and extend it by providing robust evidence indicating that conversational nature plays an important additional role. To do so, we compare information allegedly stemming from ChatGPT with information allegedly stemming from Alexa or from Wikipedia. These applications differ on the one hand in the modality in which information is presented—ChatGPT and Wikipedia are text-based, so information is read, Alexa is voice-based, so information is listened to. On the other hand, they also differ in whether they present information in a conversation-like, dynamic style (Alexa and ChatGPT) or not (Wikipedia). We furthermore assessed several potential mediators to get first hints on why these differences might occur, that is, on the likely underlying mechanisms. By also using unbranded versions in Experiment 2, we demonstrate the generalizability of our findings to dynamic text-based agents, voice-based agents, and static texts beyond ChatGPT, Alexa, and Wikipedia.

Auditorily presented information is perceived as more credible than textually presented information^8,10,11^. One reason for this *modality effect* may be that the permanence of visually presented text allows it to be reread, while auditorily presented information is typically more transient and listened to only once. Based on this logic, at least for inaccurate information, perceived information credibility should be higher when information is received from a voice-based agent like Alexa compared to both, a static text-based online encyclopedia like Wikipedia and a dynamic text-based LLM-powered agent like ChatGPT^12^.

However, voice-based output is also inherently *conversational* (i.e., interactive and agentic), which may trigger people to apply social heuristics that had evolved to guide human–human conversations to voice-based agents. Similarly, text-based LLM-powered agents typically respond to enquiries in a conversation-like manner, where one can watch them dynamically “type” the answer in real time. As a result, both voice-based and dynamic text-based agents may trigger people to apply interaction- and agency-based credibility heuristics and/or human–human communication scripts such as Grice’s conversation maxim of quality^13^ stating that in conversational settings, one tries to be truthful, resulting in increased credibility judgements^9^. Following this line of thought, both voice-based agents and dynamic text-based agents can be expected to be perceived as more credible than static text-based online encyclopedia entries because of the conversational nature in which information is presented.

Generally, because credibility judgements are particularly relevant for discerning between accurate and inaccurate information^14–16^, in the present work, we did not solely focus on an expected main effect of presentation mode on credibility judgments, but also on the moderating effect of information accuracy. Overall, we expected to find stronger effects of the presentation mode when information was partially inaccurate. Because both types of text-based presentation modes (i.e., the dynamic text-based agent and the static text-based online encyclopedia) provide the opportunity to reread the output, we expected that they would make it easier to spot inaccuracies. Thus, we predicted that differences in credibility between accurate and partially inaccurate information would be least pronounced for voice-based agents. For Experiment 2, building on exploratory findings from Experiment 1, we additionally predicted that differences in credibility between accurate and partially inaccurate information (i.e., discernment) would be less pronounced for the dynamic text-based agent than for the static online encyclopedia.

Aiming to illuminate likely underlying mechanisms, that is, *why* credibility would differ between the presentation modes, we identified two fundamental cognitive processes (processing fluency and elaborate processing) and two social-affective processes (social presence and enjoyment) from the literature that may mediate the predicted differences in credibility judgments. Both cognitive processes align well with the idea of a modality effect (read vs. listened-to) while both social-affective processes align well with the notion of a conversational effect (agentic vs. non-agentic).

Processing fluency refers to the ease with which people can process a specific message^17^. For instance, processing fluency is higher when less complicated words are used, when text is presented in a large font, and/or with high contrast^18,19^. Importantly, the more fluently information is processed, the higher is the perceived credibility^20^. Comparing modalities, written text has been argued to result in higher processing fluency than speech because people can reread it. In line with this, prior research demonstrated that processing fluency is higher for text-based agents than for voice-based conversational agents^21^. Thus, we expected that processing fluency would be higher for both text-based information search applications compared to the voice-based agent and explored also whether processing fluency mediated the effect of presentation mode on credibility.

Relatedly, dual processing models distinguish between an elaborate (or systematic) way of processing information and a peripheral (or heuristic) way of processing information^22,23^. Because information presented by voice is usually heard only once, whereas text is displayed longer and can be reread, we assumed that it is easier to process text-based information in an elaborate way, which should, in turn, decrease credibility assessments at least for partially inaccurate information. Therefore, we expected that elaborate processing would be higher for both text-based information search applications compared to the voice-based agent and aimed to also test whether elaborate processing mediated the effect of presentation mode on credibility.

Regarding social-affective processes, we looked at the potential roles of social presence and enjoyment in driving the effect of information presentation on credibility. Social presence, initially defined as the salience of other interaction partners^24^, has more recently also been applied to social agents such as robots and reflects the degree to which people perceive that they are interacting with an intelligent human being^25^. Voice-based delivery^10,26,27^ and conversational nature more generally^28^ both increase social presence, which, in turn, leads to higher credibility^29^. Therefore, we predicted that social presence would be higher for both conversational agents compared to the online encyclopedia and tested whether social presence mediated the effect of presentation mode on credibility.

Finally, an affective process that may play a role is perceived enjoyment, that is, the impression that using a device is enjoyable regardless of its functionality^30^. Empirical findings overall support the idea that conversational agents trigger higher enjoyment than static text. For instance, a qualitative study investigating voice assistant usage revealed that people turn to their voice assistants more than to traditional interfaces when they seek enjoyment^31^ and that the technology being novel contributes to this enjoyment^32^. Searching for information with ChatGPT was also demonstrated to result in higher enjoyment and satisfaction than searching for information with Google^33,34^. Thus, we expected higher enjoyment in response to the two conversational agents compared to the online encyclopedia. We also explored whether enjoyment mediated the effect of presentation mode on credibility.

LLM-based agents are widely discussed in the media and many if not most of our participants were likely to have used at least one of the information search applications investigated here before. Therefore, their opinions about the applications’ global trustworthiness may not have been neutral prior to the experiment. Because perceived trustworthiness of the information source is considered a key determinant of perceived information credibility^35^, it may impact credibility assessments in response to specific information provided by the applications. Thus, we additionally included an unbranded condition (a dynamic text-based agent, a voice-based agent, or a static text-based online encyclopedia) in Experiment 2 to explore whether branding matters. Furthermore, we globally assessed how trustworthy participants found each branded (Experiment 1) or unbranded (Experiment 2) application and descriptively compared the pattern received for the global trustworthiness assessment with the pattern for the specific credibility assessments.

**Figure 1:**
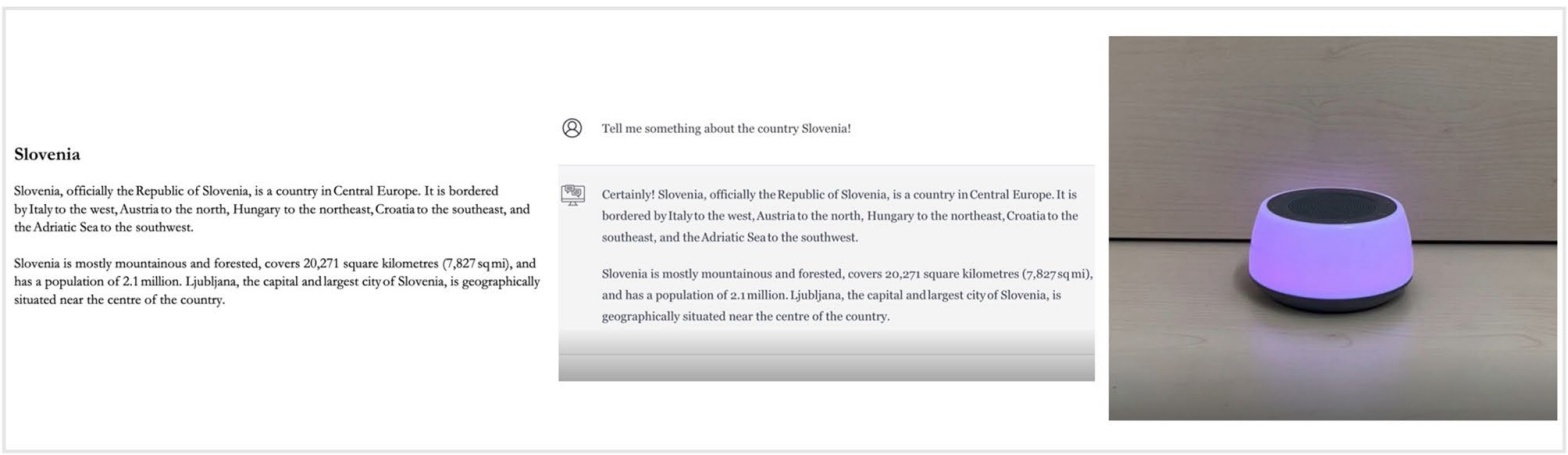
Screenshots of unbranded application mock-ups. Static text-based online encyclopedia (left), dynamic text-based LLM-powered chatbot typing out the answer, voice-based assistant; information content was identical for all branded and unbranded applications within each experiment.

To address the question of how the presentation mode influences credibility judgments and to identify likely underlying mechanisms resulting in these differences, we conducted two preregistered experiments with a total of 1222 human participants. In both experiments, upon providing written informed consent, participants were first asked about their general information search behavior in everyday life. Then, they received information about several topics related to general knowledge (one topic per page; order of topics was randomized). This information was ostensibly provided by one of three applications—a dynamic text-based agent, a voice-based agent, or a static text-based online encyclopedia (random assignment). These looked or sounded as similar as possible to the respective real applications; for the dynamic text-based agent we used videos of screencasts typing out the responses. For half the topics (set counterbalanced), the information was entirely accurate, while for the other half, the information contained several factual inaccuracies and/or internal inconsistencies (i.e., a piece of information within a snippet contradicted another piece of information provided within the same snippet); both error types are known to happen regularly during typical usage of LLMs^36^. Otherwise, the information content was identical for all applications. For Experiment 1, the application was always branded (ChatGPT, Alexa, and Wikipedia, respectively), whereas for Experiment 2, we randomly assigned participants to either a branded or an unbranded information search application (for screenshots of the unbranded application mock-ups, see Fig. 1). Participants did not interact with the applications themselves, but merely observed/listened to the information provided passively. Participants were asked to individually rate each information excerpt for its credibility by stating the extent to which they perceived the information to be accurate, trustworthy, and believable^37^ on the page following the individual topics using 7-point Likert-type rating scales. Mediators (social presence, enjoyment, perceived elaborate processing, and processing fluency) were assessed globally, that is, after rating the last information snippet for credibility. Social presence was measured using three items, e.g., “How much did you feel you were interacting with an intelligent being while reading the information/listening to the information”^25^. Participants were also asked to state how entertaining, enjoyable, and pleasant they found reading or listening to the information^31^. To assess perceived elaborate processing, participants indicated how much mental/perceptual activity they put into the task^38^ on a slider scale ranging from 0 to 100. Processing fluency was measured by asking participants the extent to which the information was comprehensible, easy to understand, clear to understand, and effortful to understand^39^. Apart from elaborate processing, all putative mediators were assessed on 7-point Likert-type rating scales. Finally, we asked how trustworthy participants perceived different information sources to be, whether they had searched online for the information presented, and asked participants to report on basic socio-demographic information. Participants were then redirected to Prolific and paid for their participation.

## Results

### Effects of presentation mode, information accuracy, and branding on credibility assessment

**Figure 2:**
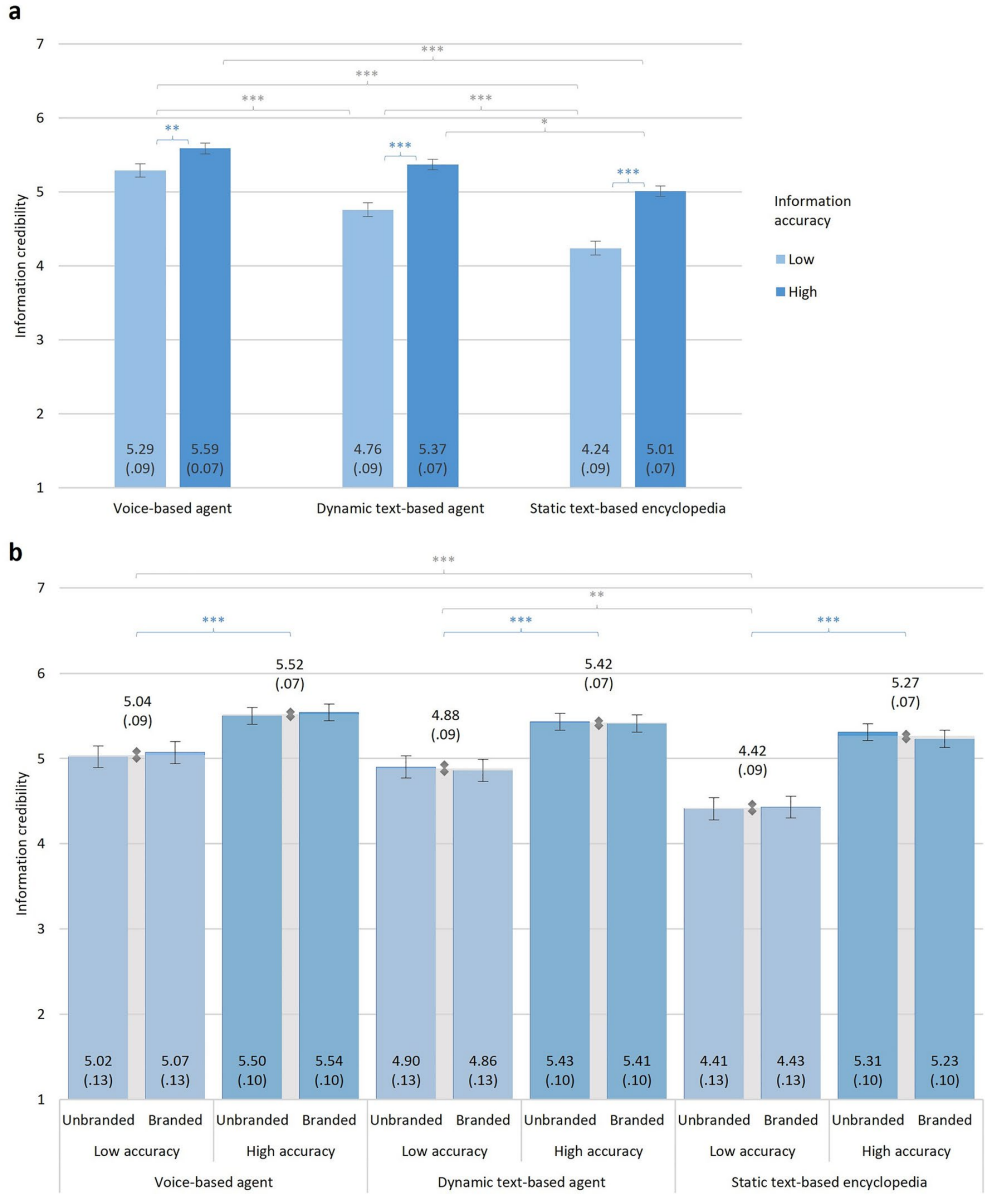
Information credibility by presentation mode, information accuracy, and branding. Panel (**a**) shows the main results of Experiment 1: Presentation mode: *F*(2, 550) = 32.10, *p* < 0.001, η^2^_partial_ = 0.11, information accuracy: *F*(1, 550) = 152.41, *p* < 0.001, η^2^_partial_ = 0.22, presentation mode × information accuracy: *F*(2, 550) = 9.36, *p* < 0.001, η^2^_partial_ = 0.03. Panel (**b**) shows the main results of Experiment 2: Presentation mode: *F*(2, 659) = 10.25, *p* < 0.001, η^2^_partial_ = 0.03, information accuracy: *F*(1, 659) = 39.36, *p* < 0.001, η^2^_partial_ = 0.06, branding: *F*(1, 659) = 0.001, *p* = 0.973, η^2^_partial_ < 0.01, presentation mode × information accuracy: *F*(2, 659) = 5.89, *p* = 0.003, η^2^_partial_ = 0.02, presentation mode × branding: *F*(1, 659) = 0.09, *p* = 0.911, η^2^_partial_ < 0.01, presentation mode × information accuracy × branding: *F*(2, 659) = 0.16, *p* = 0.855, η^2^_partial_ < 0.01. Bars represent estimated marginal means (exact values displayed within each bar), error bars show standard errors (exact values within parentheses). Post-hoc pairwise comparisons of significant two-way interaction (presentation mode × information accuracy) with Bonferroni adjustment. ***p < 0.001, **p < 0.01, *p < 0.05 (two-tailed). Blue asterisks and brackets indicate significant differences in information credibility between low and high accuracy information within a given presentation mode. Gray asterisks and brackets indicate significant differences in perceived information credibility between two modes of presentation.

We tested effects of presentation mode, information accuracy, and branding (Experiment 2 only) on credibility assessment in mixed-design analyses of variance (ANOVAs). The ANOVAs additionally included the factors topic and set (in Experiment 2, set was entered as a covariate). Main results are depicted in Fig. 2, detailed ANOVA results for Experiment 1 in Table S1, descriptive results related to effects of set and topic for Experiment 1 in Table S2, detailed ANOVA results for Experiment 2 in Table S3, and descriptive results related to effects of set and topic for Experiment 2 in Table S4 of the online supplemental materials.

As expected, credibility assessments were overall higher for accurate than for partially inaccurate information. In line with our predictions, we also found that presentation mode influenced credibility assessments in both experiments, with significantly higher credibility for the voice-based agent than the static text-based online encyclopedia. Additionally, in Experiment 1, credibility assessments were significantly higher for the voice-based agent than for the dynamic text-based agent, whereas this difference was not significant in Experiment 2.

As predicted, the main effects of accuracy and presentation mode on credibility were qualified by an interaction effect between the two. In line with our predictions, follow-up tests robustly revealed that individuals were less successful in distinguishing between accurate and partially inaccurate information when it was presented by a voice-based agent than when it was presented as static text (Experiment 1: *p* < 0.001, Experiment 2: *p* = 0.008). In Experiment 1, we additionally observed that discernment was lower when information was presented by a voice-based agent than when it was presented by a dynamic text-based agent (*p* = 0.02), while it did not differ significantly between the dynamic text-based agent and the static text condition here (*p* = 0.212). In contrast, in Experiment 2, we found that discernment was lower when information was presented by a dynamic text-based agent compared to as static text (*p* = 0.029), while it was not significantly different between the voice-based agent and the dynamic text-based agent here (*p* = 0.665). Importantly, branding did not significantly moderate the effect of presentation mode on perceived information credibility. Multi-level models with random intercepts for participants and topics that we conducted as a robustness test produced fully consistent results (main effects for mode and accuracy, interaction effect between presentation mode and accuracy; all *p*s ≤ 0.001; no significant main or interaction effect involving branding (all *p*s > 0.73).

### Putative underlying mechanisms: cognitive and social-affective processes

The descriptive results for each potential cognitive and social-affective process for both experiments are displayed in Table 1. Regarding cognitive processes, for processing fluency, results were fully in line with our predictions (see online supplemental materials; Table S5 for ANOVA results of both experiments and Tables S6 and S7 for post hoc comparisons of Experiments 1 and 2, respectively): As expected, across both experiments, we observed that processing fluency was higher when information was provided by either the dynamic text-based agent or presented as static text compared to by the voice-based agent, whereas the text-based conditions did not significantly differ. In contrast, elaborate processing did not significantly differ between conditions in either experiment, contrasting our predictions.

**Table 1:** Descriptive results for cognitive and social-affective processes in Experiments 1 and 2.

Presentation mode	*N*	Processing fluency		Elaborate processing		Social presence		Perceived enjoyment	
		*M*	*SD*	*M*	*SD*	*M*	*SD*	*M*	*SD*
Experiment 1									
Voice-based agent	183	5.61	1.06	86.85	16.24	3.41	1.97	4.60	1.64
Dynamic text-based agent	184	5.95	0.87	85.11	16.79	3.72	1.86	4.96	1.37
Static text-based encyclopedia	189	5.97	0.82	85.89	16.56	2.49	1.58	5.24	1.22
Experiment 2									
Voice-based agent	224	5.45	1.13	77.50	20.98	3.26	1.79	4.32	1.67
Dynamic text-based agent	216	5.77	0.94	75.20	23.42	3.53	1.89	4.99	1.36
Static text-based encyclopedia	226	5.90	0.87	72.83	21.88	2.56	1.57	4.53	1.67

Regarding social-affective processes, as expected, presentation mode impacted social presence, with lower perceived social presence for static text-based online encyclopedia entries compared to both voice-based agents and dynamic text-based agents. Contrary to our predictions, people felt higher enjoyment when information was presented as static or dynamic text compared to the voice-based agent, while the two text-based conditions did not significantly differ. In Experiment 2, we expected to replicate this pattern of results but found that people also felt higher enjoyment with the dynamic text-based agent than the static text.

**Figure 3:**
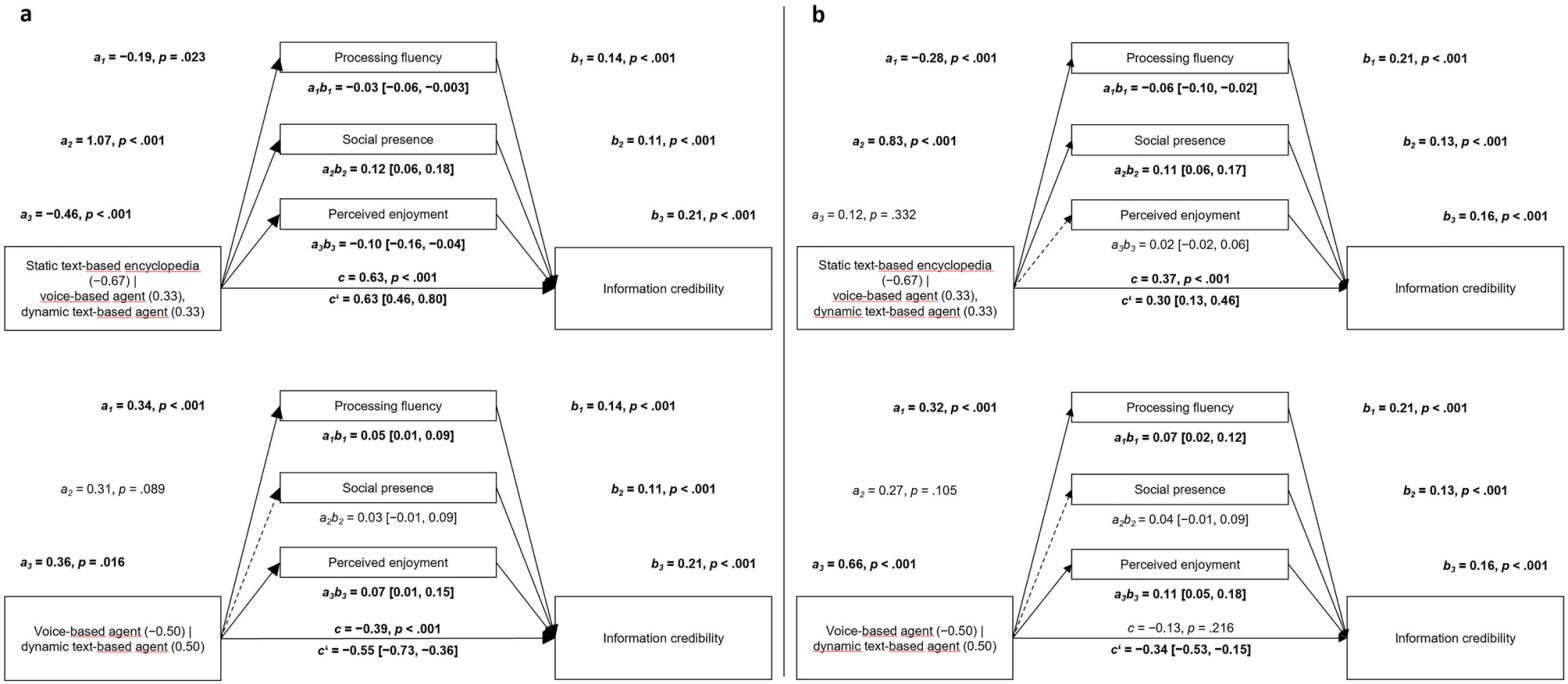
Parallel multiple mediator models predicting information credibility. Panels (**a**) and (**b**) show the results of Experiments 1 and 2, respectively. The upper part shows the comparison between static text-based encyclopedia vs. voice-based agent and dynamic text-based agent. The lower part shows the comparison between voice-based agent and dynamic text-based agent. Significant effects are displayed in bold. 95% confidence intervals of relative indirect and direct effects are displayed in square brackets.

As preregistered, we then tested for mediation effects for all expected processes for which the bivariate analyses had revealed a significant effect of presentation mode (i.e., processing fluency, social presence, and perceived enjoyment). In both experiments, processing fluency, social presence, and perceived enjoyment all significantly predicted credibility perceptions (Fig. 3).

Regarding the first comparison (static text-based encyclopedia vs. voice- and dynamic text-based agent), in line with the bivariate results of both experiments, the conversational agents elicited higher perceived information credibility than the static text-based encyclopedia. Experiment 1 yielded significant negative presentation mode effects on processing fluency and perceived enjoyment and a positive effect on social presence, that is, compared to the static text-based encyclopedia, the conversational agents elicited lower processing fluency and perceived enjoyment, but higher social presence. Processing fluency, perceived enjoyment, and higher social presence all showed positive relationships with information credibility. Experiment 2 also yielded a negative effect of presentation mode on processing fluency and a significant positive effect on social presence (but not perceived enjoyment), that is, compared to the static text-based encyclopedia, the conversational agents elicited lower processing fluency, but higher social presence. Again, processing fluency, social presence, and perceived enjoyment all showed positive relationships with information credibility. Significant relative indirect effects were found via processing fluency, social presence, and perceived enjoyment in Experiment 1 and via processing fluency and social presence in Experiment 2. Including the mediators in the model did not lead to a substantial reduction of the relative direct effect of application on perceived information credibility in either experiment, possibly because the positive and negative indirect effects cancelled each other out.

Regarding the second comparison (voice- vs. dynamic text-based agent), the dynamic text-based agent elicited lower credibility perceptions than the voice-based agent in Experiment 1, whereas this effect was not significant in Experiment 2. The dynamic text-based agent led to higher processing fluency and perceived enjoyment in both studies. The effects on social presence were not significant. Like in the first comparison, processing fluency, perceived enjoyment, and higher social presence all showed positive relationships with information credibility. Both experiments yielded significant relative indirect positive effects via processing fluency and perceived enjoyment. Compared to the direct effect, the total effect was smaller in size (i.e., less negative). Thus, mediators partially suppress the negative direct effect. In Experiment 2, the indirect effects fully suppress the negative direct effect, rendering a significant negative direct effect into a non-significant total effect.

### Global trustworthiness of the applications

**Figure 4:**
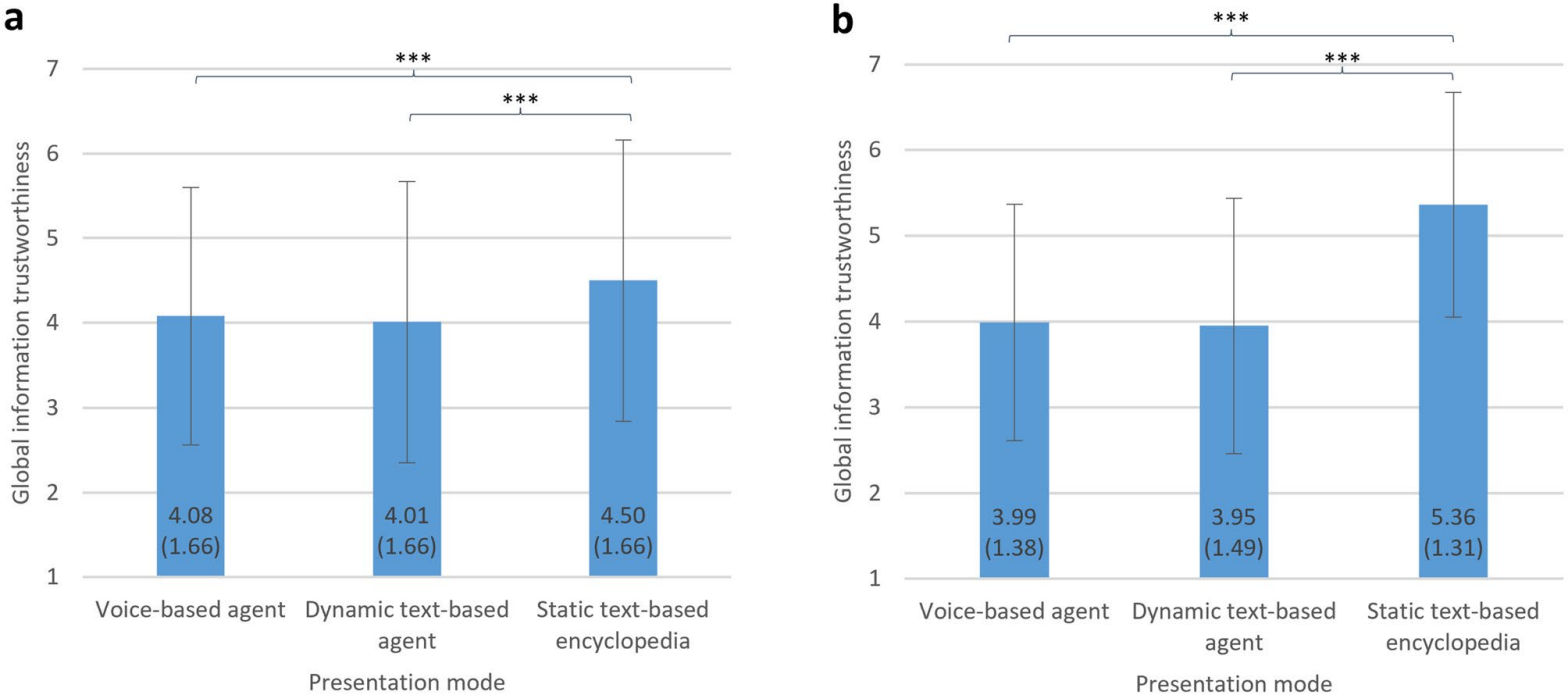
Global trustworthiness. Results of Experiment 1 are displayed in Panel (**a**), results of Experiment 2 in Panel (**b**). Bars represent means (exact values displayed within each bar), error bars show standard deviations (exact values in parentheses). *** *p* < 0.001 (two-tailed).

We showed that information provided by voice- or dynamic text-based agents is perceived as more credible than information provided by a static-text based online encyclopedia and that discernment is lower for these as well. However, exploratory analyses yielded an interesting discrepancy between perceived information credibility when being exposed to actual information and global trustworthiness ratings regarding the three information search applications. Here, online encyclopedias were rated as most trustworthy, while no significant differences were observed between voice-based and dynamic text-based agents (Fig. 4).

## Discussion

With recent advances in LLMs, people increasingly turn to conversational agents like ChatGPT, Bing AI, or Google Bard when searching for information and LLMs are increasingly implemented into voice-based agents like Alexa as well. However, concerns have been raised about their oftentimes factually inaccurate responses. Our findings here suggest that this may be particularly problematic because the conversational nature in which information is delivered by these agents decreases people’s readiness to discern between accurate and inaccurate information, even when text-based presentation would allow them to reread the information more than once.

The present findings provide robust evidence that information received by dynamic text-based agents like ChatGPT is perceived as more credible than identical information presented as static text, but as less (Experiment 1) or equally (Experiment 2) credible compared to the same information presented by voice-based agents. These effects were strongly driven by differences in discernment between accurate and inaccurate information, which is, arguably, highly relevant^14–16^, and importantly complement previous research showing similar findings for actual information search that were however observed in a much less controlled setting^34^ (because the information content itself differed). The present research also makes important theoretical contributions^40^ by demonstrating that the observed effects were independent of specific branded applications and even showed opposite pattern to people’s self-reported general assessments of the applications’ trustworthiness.

The most plausible interpretation for the observed pattern of results appears to be that both a modality effect (i.e., reading vs. listening) and an effect of conversational nature (i.e., conversational vs. non-conversational) work in parallel and in partially opposing ways: discernment between accurate and inaccurate information benefitted from reading (vs. listening) and from being presented in a non-conversational (vs. conversational) way. Because dynamic text-based agents combine both, higher discernment through reading and reduced discernment through the conversational nature, they score between voice-based agents (lower discernment through listening and conversational nature) and static text (higher discernment through reading and non-conversational nature). Mediation analyses overall corroborated this interpretation in suggesting that when comparing the two conversational agents with the static text-based encyclopedia, the positive and negative effects of the different cognitive and social-affective processes seemed to cancel each other out. Interestingly, when comparing the conversational agents with each other, processing fluency and perceived enjoyment seemed to partially (Experiment 1) or fully (Experiment 2) suppress the effect of presentation mode on credibility because the effect got stronger when accounting for the indirect effects. Notably, all reported significant effects were small to medium in size and thereby exceeded our a priori defined threshold for a minimal effect size of interest (SESOI). These mediation results should nevertheless be interpreted with caution because the effect of enjoyment was opposite to what we had predicted and because non-experimental mediation does not allow for causal interpretation^41–44^. As such, the identified putative mechanisms should be seen as setting a direction for future experimental work rather than a final conclusion. For instance, the effects of processing fluency could be experimentally investigated by adapting the fonts of the text-based conditions to be less easily readable. Similarly, social presence could be made more salient for the static text condition by reminding participants that the snippets had been written by humans or reduced by making the voice sound more mechanical.

This work has important real-world implications. Many users have misconceptions about LLMs and think they work like search engines^45,46^. Since more and more search engines integrate LLM-based chatbots into their services (e.g., Bing, Google), the danger is high that people will be even less able to differentiate between the different ways of information generation if no targeted effort is taken to prevent this. Even though several LLMs present a disclaimer that not all information may be correct, it is unclear to what degree being aware of this risk is enough especially when information is presented in a conversational style. Indeed, our participants reported lower general trust in information received by text-based or voice-based agents than in information received by an online encyclopedia and still found the specific pieces of information more credible when they were presented by one of these agents. Algorithm literacy should thus not only address issues such as hallucinations or biases but also knowledge about information processing in different modalities such as reading versus listening. Similarly, in addition to making efforts to specifically highlighting potentially incorrect or misleading information in the LLM responses, which has shown promise in prior work^34^, technology companies may be advised to decrease the degree to which interactions with their technologies feel human-like during information search^47,48^. Indeed, recent research suggests that there are no differences in perceived credibility of information between Wikipedia, ChatGPT, and an unbranded, raw text interface when the conversational nature of ChatGPT is made less salient^12^.

A limitation of the present research is that participants did not interact with the information search applications themselves but rather passively viewed or listened to another person’s ostensible interactions with them. It is therefore impossible to conclude with certainty that the same patterns of results would be observed for real interactions. This may partially explain the rather counterintuitive findings regarding enjoyment, with, at least descriptively, lowest enjoyment for the voice-based agent in both experiments. Similarly, participants were presented with information related to topics that may not be what they would naturally look for, even though we tried to cover a wide range of topics. To what degree topic relevance and/or familiarity impacts the observed effects of information presentation mode will be an important question for future research. However, we would argue that the observed modality effect (listening versus reading) on perceived credibility should not be heavily affected by this. In contrast, the effect of the conversational nature (dynamic agents versus static text) on credibility may be less pronounced in the artificial setting of an online experiment compared to direct interaction. However, the observed effect of the conversational nature (i.e., agency and interactivity) on credibility should, arguably, be stronger rather than weaker in a more interactive setting, especially because experiences of social presence, which mediated the effect, may be assumed to get stronger with increasing familiarity with the agent.

Moreover, we would like to stress that because our proposed mediators were not independently manipulated, the present work does not allow for the conclusion that they are indeed the causal drivers of the observed effects of presentation mode on credibility^41–44^. Much like for other correlational work, it is impossible to rule out that the observed correlational effects are driven by another, potentially unmeasured factor. Combined with our experimental findings addressing the how, findings from our mediation analysis may, however, be helpful in identifying fruitful directions for future research addressing the why. To establish causality, future research will need to experimentally manipulate mediators to test their causal effects on credibility.

Finally, we kept information constant between the three applications. While this allowed us to conduct a more stringent test of effects of presentation mode on credibility and the potentially underlying mechanisms and thereby increased the internal validity of our research, this experimental design choice naturally decreases external validity^49^. However, again we believe that this should primarily affect the effect of conversational nature on credibility in a way that agents (voice-based and text based) differed less from the static text-based application in terms of language-based agency and interactivity cues, which have, themselves, been shown to increase credibility when facing inaccurate information^34,48^. Taken together, we therefore believe that, if anything, the present findings underestimate the true effect of conversational nature on credibility.

It appears safe to say that LLMs are here to stay and that newer versions and implementations into voice-based agents will likely make interactions with LLM-powered agents feel even more rather than less conversational and human-like. LLMs are designed to generate the most likely sequences of words, not to optimize or assess the accuracy of their own output^50^, and are, in consequence, known to regularly produce inaccurate output^3–5^. It is, therefore, of utmost importance to understand how presentation mode impacts credibility assessment. Our findings indicate that a conversational style poses a threat to adequate credibility judgments when facing inaccuracies whereas textual presentation promotes them.

## Methods

### Experiment 1

#### Design and participants

We employed a 3 (presentation mode: static text-based encyclopedia vs. dynamic text-based agent vs. voice-based agent; between-subjects factor) × 2 (information accuracy: low vs. high; within-subjects factor) × 2 (set: A vs. B) mixed factorial experimental design. After providing written informed consent, participants were randomly assigned to one of the three presentation modes. In each condition, we presented participants with six questions and answers that varied in accuracy. We counterbalanced the low and high accuracy answers by randomly assigning participants to one of two sets: low accuracy answers in set A (low: Appendicitis, Bones, Wolf) were presented with a high accuracy answer in set B (low: Slovenia, Hookah, Titanic) and vice versa. Detailed absolute cell sizes for both Experiments 1 and 2 are reported in Table S8 of the online supplemental materials. The study received ethics approval from the IRB of the first author’s institution (Ethics Committee of the Leibniz-Institut für Wissensmedien). We preregistered the study on the Open Science Framework (OSF) platform: https://osf.io/puyv7.

We based our sample size considerations on prior work^8^ which analyzed data from *N* = 300 participants. Because our experiment included the additional between-subjects factor set and because we wanted to test more and higher-level interaction effects, we decided to collect more observations^51^. We recruited *N* = 600 UK-based participants via Prolific in June 2023. Participants had to be at least 18 years old, and fluent in English. They received 2.25 GBP for taking part in the experiment. Following our preregistration, we excluded participants who searched for the presented information online while completing the experiment (*n* = 44); fact-checking did not differ between presentation modes (χ2 = 1.52, *p* = 0.47). The final sample comprised *N* = 556 participants (*n*_female_ = 281, *M*_age_ = 43.33, *SD*_age_ = 13.10). Most participants (*n* = 348) had studied or were currently studying at a higher education institution. One hundred and eighty-nine participants were assigned to the static text-based encyclopedia condition. The voice- and dynamic text-based agent conditions comprised 183 and 184 participants, respectively. Two-hundred and eighty-one participants saw Set A and 275 participants saw Set B.

#### Procedure

Once they had started the questionnaire, participants were first asked about their information search behavior. Then, they were presented with six information snippets, each consisting of a question and an answer. Participants were asked to rate the credibility of the information in each snippet. After participants had read or listened to all information snippets, we assessed the potential mediators. In addition, we asked how involved participants were in the topics, how trustworthy they perceived different information sources to be, and whether they had searched online for the information presented. Participants were then redirected to Prolific and paid for their participation.

#### Stimuli and manipulation of independent variables

To prepare the information snippets, we selected six questions that related to general knowledge and covered diverse topics^8^: What do I do when I encounter a wolf? What are the risks of hookah smoking? How many people died when the Titanic sank? What is appendicitis? How many bones are in the human body? Tell me something about the country Slovenia! For each of them, we first created the accurate answers (high accuracy condition) by shortening the respective Wikipedia articles and informational texts from additional, trustworthy sources. To create the snippets for the low accuracy condition, we used the same, short answers, but built in false information. To make sure that detecting inaccuracies did not entirely depend on prior knowledge, we included logical inconsistencies in addition to factual errors. For instance, the answer to the question of how to behave when encountering a wolf included the correct recommendation to make loud noises, combined with the inconsistent reason to do so in order not to be noticed. Both error types occur regularly in the context of everyday LLM use^36^. The accuracy of the presented information was manipulated by the absence or presence of factually or logically false information. High accuracy answers did not contain false information. Low accuracy answers contained two to four errors.

We manipulated presentation mode by creating snippets of information that looked or sounded as similar as possible to the real-life applications Wikipedia (static text-based encyclopedia), ChatGPT (dynamic text-based agent), and Alexa (voice-based agent). Each application gave the same answer in terms of content. In the static text-based encyclopedia condition, participants were asked to read the answers, allegedly stemming from Wikipedia. The Wikipedia screenshots were created by manipulating the local HTML source code of the relevant Wikipedia article and inputting the desired text into the article section. In the dynamic text-based agent condition, we asked participants to follow six screencasts, each showing a user asking ChatGPT a question and ChatGPT typing the answer to that question. Participants did not interact with the applications themselves, but merely observed/listened to the information provided passively. The snippets were created using Open AI’s (large) language model ChatGPT Version March 14 and the output prompts were collected between March 28th, 2023, and March 30th, 2023. In the voice-based agent condition, participants were asked to watch six videos of a user asking Alexa a question and Alexa answering this question. The voice files were created with the skill “texttovoice.io” function from the Amazon Alexa application. Then, Alexa was videotaped vocalizing the same content that was used in the other conditions. We minimized ambient noise and improved video quality by using bright light in the recordings. We added all materials to our OSF repository: https://osf.io/5dqxe/.

#### Measures

We used an adapted *information credibility* scale^37^ which had three items (accurate, trustworthy, believable) that were answered on Likert-type rating scales ranging from 1 = *describe it very poorly* to 5 = *describe it very well* (α = 0.63).

*Social presence* was measured with a 7-point Likert-type rating scale from 1 = *not at all* to 7 = *very*^52^. Question wording depended on whether the information was provided as audio or text, e.g., “How much did you feel you were interacting with an intelligent being while reading the information/listening to the information?” (α = 0.96).

To measure *elaborate processing*, we used the two items “Please indicate how much mental/perceptual activity you put in the task?” from the NASA-TLX task^38^ as a slider scale (0 = *none* to 100 = *full*) (Spearman-Brown ρ = 0.83).

Our adapted *processing fluency* scale^39^ consisted of the four items “To which extent was the information comprehensible/easy to understand/clear to understand/effortful to understand?”. Items were answered on a 7-point Likert-type rating scale from 1 = *not at all* to 7 = *very* (α = 0.66).

To assess participants’ *perceived enjoyment* while reading or listening to the information, we adapted three items^31^ such as “I found reading the information / listening to the information entertaining”, to be answered on 7-point Likert-type rating scales (1 = *strongly disagree* to 7 = *strongly agree*) (α = 0.95).

Table S9 of the online supplemental materials shows the means, standard deviations, and Cronbach’s α as well as the bivariate correlations between the primary continuous variables for both experiments.

In addition to the specific credibility assessments, we used a single item to measure the trustworthiness of specific sources, e.g., Wikipedia, Alexa, and ChatGPT, respectively. Participants rated the item “How trustworthy do you find information from the following sources?” using a 7-point Likert-type rating scale from 1 = *not trustworthy at all* to 7 = *very trustworthy.* Additionally, a *can’t judge* option was included.

To ascertain whether participants looked up the information during the experiment, we posed the question: “While participating in the study, did you check facts online about the information presented?” with options for *yes* or *no* responses. In addition, we measured participants’ search behavior and topic involvement, but these variables were not used in the analyses. In Experiment 1, we additionally explored the role of social attraction (only for conversational agents), but we had no specific predictions. Results regarding this measure are presented in Table S10 of the online supplemental materials.

#### Statistical analysis

We conducted a mixed design ANOVA with repeated measures on our data: 3 (presentation mode: dynamic text-based agent vs. voice-based agent vs. static text-based encyclopedia) × 2 (set: A and B) × 2 (accuracy: low and high) × 3 (topic, referring to the order in each set). We categorized presentation mode and set as between-subjects factors, whereas accuracy and topic formed within-subjects factors. To investigate the processes underlying the relationship between presentation mode and perceived information credibility, a parallel multiple mediator model was estimated using PROCESS model 4 with 10,000 bootstrap samples^53^. We included presentation mode as a Helmert-coded independent variable by first comparing the static text-based encyclopedia to the voice-based agent and the dynamic text-based agent using the following coding scheme: –2/3 (static text-based encyclopedia), + 1/3 (voice-based agent), + 1/3 (dynamic text-based agent). We then compared the voice-based agent to the dynamic text-based agent using the coding scheme 0 (static text-based encyclopedia), –1/2 (voice-based agent), + 1/2 (dynamic text-based agent).

### Experiment 2

#### Design and participants

We employed a 3 (presentation mode: dynamic text-based agent vs. voice-based agent vs. static text-based encyclopedia; between-subjects-factor) × 2 (information accuracy: low vs. high; within-subjects factor) × 2 (branding: branded vs. unbranded; between-subjects factor) × 2 (set A vs. B, between-subjects factor) mixed factorial experimental design. After providing written informed consent, participants were randomly assigned to one of six combinations of presentation mode and branding. In each condition, we presented participants with four questions and answers that varied in accuracy. We counterbalanced the low and high accuracy answers by randomly assigning participants to one of two sets: Low accuracy answers in set A (low: Appendicitis, Hookah) were presented with high accuracy answers in set B (low: Slovenia, Titanic) and vice versa (see Table S8 of the online supplemental materials for detailed absolute cell sizes). The study received ethics approval from the IRB of the first author’s institution (Ethics Committee of the Leibniz-Institut für Wissensmedien) and all methods were performed in accordance with the relevant guidelines and regulations. We preregistered the study on the Open Science Framework (OSF) platform: https://osf.io/yps8k.

We defined a smallest effect size of interest (SESOI) for all hypotheses and research questions, including the a and b paths in the mediation models, a priori. A SESOI of *d* = 0.20/Pearson’s *r* = 0.10 seemed plausible according to meta-analytical effect sizes in communication research and social psychology and Cohen's benchmarks. Thus, everything below we deemed to be practically irrelevant, even if significant. We based our sample size considerations on an apriori safeguard power analysis^54^ for the effect of presentation mode on perceived information credibility. Considering the results of Experiment 1, the smallest contrast was between Alexa and ChatGPT (voice vs. dynamic text): *d* = 0.31. Based on a safeguard power analysis using the lower bound of a 60% CI^54^, *d* = 0.24, a significance level of α = 0.05, and a power = 0.80, a sample size of 648 was required. Similar to Experiment 1, we expected around 10% of participants to search for information online while taking the experiment. Because we intended to exclude those participants, we recruited a total of *N* = 716 participants. Participants from the UK with a minimum age of 18 and fluent in English were recruited via Prolific in October 2023. They received 2.25 GBP for taking part in the experiment. Following our preregistration, we excluded participants who reported that they had searched for the presented information online (*n* = 50); again, fact-checking did not differ between presentation modes (χ^2^ = 0.69, *p* = 0.71). The final sample comprised *N* = 666 participants (*n*_female_ = 330, *M*_age_ = 41.96, *SD*_age_ = 13.28). Most participants (*n* = 412) had studied or were currently studying at a higher education institution. Two hundred and twenty-six participants were assigned to the static text-based encyclopedia condition. The voice-based and dynamic text-based agent conditions comprised 224 and 216 participants, respectively. Three-hundred and thirty-four participants saw a branded and 332 participants saw an unbranded presentation mode. Three-hundred and thirty-four participants saw Set A and 332 participants saw Set B.

#### Stimuli and manipulation of independent variables

Stimuli for of Experiment 2 were identical to Experiment 1 with the following exceptions: The results of Experiment 1 suggested that the topic “What do I do when I encounter a wolf? “ was overall considered less credible than the other topics. In addition, the topic “How many bones are in the human body?” led to unexpected response behavior manifesting in slightly lower (vs. higher) credibility values for high (vs. low) accuracy information in the voice-based and the dynamic text-based agent conditions (see Table S2 of the online supplemental materials). We thus decided not to present those two topics to participants in Experiment 2; that is, we only included the four remaining topics Appendicitis, Slovenia, Hookah, and Titanic. Otherwise, the questions and answers for both the high and the low accuracy condition were the same as in Experiment 1.

For branded applications, the manipulation of presentation mode was identical to Experiment 1. For the unbranded conditions, we stripped the snippets of all brand-related information so that they looked and sounded like a generic voice-based agent, a dynamic text-based agent, or a static text-based encyclopedia (Fig. 1). All materials can be found in our OSF repository: https://osf.io/5dqxe/.

#### Measures

The measures correspond to the ones used in Experiment 1. The wording of the questions used to measure elaborate processing^38^ was slightly adapted compared to Experiment 1: “Please indicate how much mental/perceptual activity you put in processing the information you read/listened to?”. We used single items to measure the trustworthiness of specific sources, e.g., online encyclopedia, voice assistant, and AI-based chatbot. Participants rated the item “How trustworthy do you find information from the following sources?” using a 7-point Likert-type rating scale from 1 = *not trustworthy at all* to 7 = *very trustworthy.* Additionally, a *can’t judge* option was included.

#### Statistical analysis

As preregistered, we conducted a mixed design ANOVA with repeated measures using the following design: 3 (presentation mode: dynamic text-based agent vs. voice-based agent vs. static text-based encyclopedia) × 2 (branding: branded vs. unbranded) × 2 (accuracy: low and high) × 2 (topic, referring to the order in each set). Set was included as a covariate this time. Presentation mode and branding were between-subjects factors, whereas accuracy and topic were within-subjects factors. To investigate the processes underlying the relationship between presentation mode and perceived information credibility, a parallel multiple mediator model was estimated using PROCESS model 4 with 10,000 bootstrap samples^53^. We included presentation mode as a Helmert-coded independent variable by first comparing the static text-based encyclopedia to the voice-based agent and the dynamic text-based agent using the following coding scheme: − 2/3 (static text-based encyclopedia), + 1/3 (voice-based agent), + 1/3 (dynamic text-based agent). We then compared the voice-based agent to the dynamic text-based agent using the coding scheme 0 (static text-based encyclopedia), − 1/2 (voice-based agent), + 1/2 (dynamic text-based agent).

### Supplementary Information

Supplementary Tables.

## Data Availability

Study data and analysis code are available on our OSF repository: https://osf.io/5dqxe/.
