# Supplementary material for: Conversational presentation mode increases credibility judgements during information search with ChatGPT
Source: Sci Rep. 2024 Jul 25;14:17127. doi: 10.1038/s41598-024-67829-6 (PMC11272919; doi:10.1038/s41598-024-67829-6)
Supplement: Supplementary file 1 — Supplementary Tables. [file 41598_2024_67829_MOESM1_ESM.docx]

**Supplementary Online Materials**

for

Conversational Presentation Mode Increases Credibility Judgements During Information Search with ChatGPT

**Table S1. Detailed results of the mixed-design analyses of variance on credibility in Experiment 1**

| Source | *df* | *F* | *p* | η²_p_ |
| --- | --- | --- | --- | --- |
| **Between-Subjects Effects** |  |  |  |  |
| presentation mode | 2 | 32.10 | < .001 | .11 |
| set | 1 | 13.53 | < .001 | .02 |
| presentation mode x set | 2 | 0.11 | 0.897 | .00 |
| error (presentation mode) | 550 |  |  |  |
| **Within-Subjects Effects** |  |  |  |  |
| accuracy | 1 | 152.41 | < .001 | .22 |
| accuracy x presentation mode | 2 | 9.36 | < .001 | .03 |
| accuracy x set | 1 | 8.22 | 0.004 | .02 |
| accuracy x presentation mode x set | 2 | 1.33 | 0.265 | .01 |
| error (accuracy) | 550 |  |  |  |
| topic | 2 | 155.81 | < .001 | .22 |
| topic x presentation mode | 4 | 9.77 | < .001 | .03 |
| topic x set | 2 | 7.44 | 0.001 | .01 |
| topic x presentation mode x set | 4 | 3.99 | 0.003 | .01 |
| error (topic) | 1100 |  |  |  |
| accuracy x topic | 2 | 30.39 | < .001 | .05 |
| accuracy x topic x presentation mode | 4 | 1.39 | 0.234 | .01 |
| accuracy x topic x set | 2 | 104.00 | < .001 | .16 |
| accuracy x topic x presentation mode x set | 4 | 8.67 | < .001 | .03 |
| error (accuracy x topic) | 1100 |  |  |  |

Note. In addition to the main results reported in the text, our analysis also revealed several unpredicted interactions involving set and/or topic that can only be interpreted meaningfully when looking at the also significant three-way interaction between accuracy, set, and topic (because topic number refers to a different topic depending on set). This effect was further qualified by a significant four-way-interaction between medium, accuracy, set, and topic. Importantly, the interaction effects did not challenge our main findings/conclusions. Instead, they revealed that the main effect of presentation mode on credibility descriptively held for every individual combination of topic and accuracy-level (*M*_Alexa_ > *M*_ChatGPT_ ≥ *M*_Wikipedia_) and that the beneficial effect of high accuracy on credibility assessment was consistently higher in the Wikipedia compared to the Alexa and the ChatGPT condition (for which it even reversed for information on the topic “bones”), see Table S2.

**Table S2. Descriptive credibility ratings as a function of presentation mode, information accuracy, topic, and set for Experiment 1**

|  |  |  |  | Alexa | |  | ChatGPT | |  | Wikipedia | |
| --- | --- | --- | --- | --- | --- | --- | --- | --- | --- | --- | --- |
|  |  |  |  | ***M*** | ***SE*** |  | ***M*** | ***SE*** |  | ***M*** | ***SE*** |
| Set A | *Low* | *appendicitis* |  | 5.59 | .16 |  | 5.18 | .17 |  | 4.85 | .16 |
|  |  | *bones* |  | 5.84 | .15 |  | 5.53 | .15 |  | 4.95 | .14 |
|  |  | *wolf* |  | 4.91 | .19 |  | 3.66 | .20 |  | 3.15 | .19 |
|  | *High* | *Slovenia* |  | 5.96 | .12 |  | 5.79 | .12 |  | 5.49 | .12 |
|  |  | *hookah* |  | 5.52 | .13 |  | 5.27 | .14 |  | 4.54 | .13 |
|  |  | *Titanic* |  | 5.90 | .14 |  | 5.83 | .15 |  | 5.57 | .14 |
|  |  |  |  | ***M*** | ***SE*** |  | ***M*** | ***SE*** |  | ***M*** | ***SE*** |
| Set B | *Low* | *Slovenia* |  | 5.36 | .17 |  | 5.05 | .16 |  | 5.05 | .16 |
|  |  | *hookah* |  | 5.31 | .15 |  | 4.65 | .15 |  | 3.92 | .15 |
|  |  | *Titanic* |  | 4.74 | .20 |  | 4.47 | .19 |  | 3.52 | .19 |
|  | *High* | *appendicitis* |  | 5.60 | .12 |  | 5.36 | .12 |  | 5.32 | .12 |
|  |  | *bones* |  | 5.58 | .14 |  | 5.47 | .13 |  | 5.43 | .13 |
|  |  | *wolf* |  | 4.98 | .15 |  | 4.50 | .14 |  | 3.71 | .14 |

Note. The topic *wolf* was overall considered less credible than the other topics and the topic *bones* led to unexpected response behavior manifesting in slightly lower (vs. higher) credibility values for high (vs. low) accuracy information in the Alexa and the ChatGPT conditions. Importantly, however, the main effect of presentation mode on credibility descriptively held for every individual combination of topic and accuracy-level (*M*_Alexa_ > *M*_ChatGPT_ ≥ *M*_Wikipedia_).

**Table S3. Detailed results of the mixed-design analyses of variance on credibility in Experiment 2**

| Source | *df* | *F* | *p* | η²_p_ |
| --- | --- | --- | --- | --- |
| **Between-Subjects Effects** | | | | |
| presentation mode | 2 | 10.25 | < .001 | .03 |
| set | 1 | 57.72 | < .001 | .08 |
| branding | 1 | 0.00 | 0.973 | .00 |
| mode x branding | 2 | 0.09 | 0.911 | .00 |
| **Within-Subjects Effects** | | | | |
| accuracy | 1 | 39.36 | < .001 | .06 |
| accuracy x presentation mode | 2 | 5.89 | 0.003 | .02 |
| accuracy x set | 1 | 4.94 | 0.027 | .01 |
| accuracy x branding | 1 | 0.08 | 0.779 | .00 |
| accuracy x presentation mode x branding | 2 | 0.16 | 0.855 | .00 |
| error (accuracy) | 659 |  |  |  |
| topic | 1 | 0.80 | 0.373 | .00 |
| topic x presentation mode | 2 | 4.93 | 0.007 | .02 |
| topic x set | 1 | 21.20 | < .001 | .03 |
| topic x branding | 1 | 2.21 | 0.137 | .00 |
| topic x presentation mode x branding | 2 | 0.64 | 0.528 | .00 |
| error (topic) | 659 |  |  |  |
| accuracy x topic | 1 | 2.75 | 0.098 | .00 |
| accuracy x topic x presentation mode | 2 | 1.09 | 0.336 | .00 |
| accuracy x topic x set | 1 | 0.08 | 0.774 | .00 |
| accuracy x topic x branding | 1 | 2.98 | 0.085 | .01 |
| accuracy x topic x presentation mode x branding | 2 | 0.41 | 0.664 | .00 |
| error (accuracy x topic) | 659 |  |  |  |

Note. In addition to the main results reported in the text, our results also unveiled several unforeseen relationships related to set and/or topic. There was a significant interaction between accuracy and set, indicating that the accuracy effect is stronger in set A. There were also significant interactions between topic and presentation mode, between topic and set, and between topic and accuracy. These interactions involving topic and/or set can only be interpreted meaningfully when looking at the higher order interactions because topics 1 and 2 refer to different topics depending on the set and accuracy condition. Comparing the descriptive means (displayed in Table S4) reveals that the three interactions mainly indicate that the credibility ratings vary across topics. The partially inaccurate information for the topic Titanic was perceived as less credible than other partially accurate information snippets. The credibility of the accurate versions of the topics appendicitis and hookah in set B were lower than in Experiment 1 and the differences with the partially inaccurate versions in set A were less pronounced or even descriptively reversed. Importantly, within the sets, the pattern that accurate information is perceived as more credible than partially inaccurate information is stable, as is the finding that presentation mode matters more for the partially inaccurate information. When information was partially inaccurate, it was consistently perceived as least credible when it was presented as static text across all topics and both sets.

**Table S4. Descriptive credibility ratings as a function of presentation mode, information accuracy, topic, and set for Experiment 2**

|  |  |  | Voice | |  | Dynamic | |  | Static | |
| --- | --- | --- | --- | --- | --- | --- | --- | --- | --- | --- |
|  |  |  | ***M*** | ***SE*** |  | ***M*** | ***SE*** |  | ***M*** | ***SE*** |
| Set A | *Low* | *appendicitis* | 5.59 | .14 |  | 5.16 | .14 |  | 5.10 | .14 |
|  |  | *hookah* | 5.17 | .16 |  | 4.88 | .17 |  | 4.32 | .16 |
|  | *High* | *Slovenia* | 5.83 | .11 |  | 5.69 | .12 |  | 5.81 | .11 |
|  |  | *Titanic* | 5.78 | .11 |  | 5.71 | .11 |  | 5.73 | .11 |
|  |  |  | ***M*** | ***SE*** |  | ***M*** | ***SE*** |  | ***M*** | ***SE*** |
| Set B | *Low* | *Slovenia* | 5.08 | .14 |  | 5.15 | .14 |  | 4.70 | .14 |
|  |  | *Titanic* | 4.33 | .16 |  | 4.34 | .17 |  | 3.57 | .16 |
|  | *High* | *appendicitis* | 5.38 | .11 |  | 5.30 | .12 |  | 5.03 | .11 |
|  |  | *hookah* | 5.07 | .11 |  | 4.98 | .11 |  | 4.49 | .11 |

**Table S5. Results of the univariate analyses of variance on cognitive and social-affective processes in both experiments**

|  | Processing Fluency | | | |  | Elaborate Processing | | | | |  | | Social Presence | | | |  | Perceived Enjoyment | | | | |
| --- | --- | --- | --- | --- | --- | --- | --- | --- | --- | --- | --- | --- | --- | --- | --- | --- | --- | --- | --- | --- | --- | --- |
| Effect | *df* | *F* | *p* | η²_p_ |  | *df* | *F* | *p* | η²_p_ |  | | *df* | | *F* | *p* | η²_p_ |  | *df* | *F* | *p* | η²_p_ |  |
| Presentation Mode  **Experiment 1** | 2, 553 | 8.84 | < .001 | .03 |  | 2, 553 | 0.51 | 0.601 | .00 |  | | 2, 553 | | 23.38 | < .001 | .08 |  | 2, 553 | 9.41 | < .001 | .03 |  |
| **Experiment 2** | 2, 663 | 12.31 | < .001 | .04 |  | 2, 663 | 2.52 | 0.081 | .01 |  | | 2, 663 | | 17.86 | < .001 | .05 |  | 2, 663 | 10.93 | < .001 | .03 |  |

**Table S6. Results of post hoc comparisons for the univariate analyses of variance on cognitive and social-affective processes in Experiment 1**

| **Processing Fluency** |  | Mean Difference | *SE* | *t* | Cohen's *d* | *p*_bonf_ |
| --- | --- | --- | --- | --- | --- | --- |
| Voice-based agent | Dynamic text-based agent | -0.340 | 0.096 | -3.542 | -0.370 | 0.001** |
|  | Static text-based encyclopedia | -0.358 | 0.095 | -3.745 | -0.388 | < .001*** |
| Dynamic text-based agent | Static text-based encyclopedia | -0.017 | 0.095 | -0.180 | -0.019 | 1.000 |
| **Social Presence** | | | | | | |
| Voice-based agent | Dynamic text-based agent | -0.313 | 0.189 | -1.657 | -0.173 | 0.294 |
|  | Static text-based encyclopedia | 0.918 | 0.188 | 4.891 | 0.507 | < .001*** |
| Dynamic text-based agent | Static text-based encyclopedia | 1.231 | 0.187 | 6.568 | 0.680 | < .001*** |
| **Perceived Enjoyment** | | | | | | |
| Voice-based agent | Dynamic text-based agent | -0.357 | 0.148 | -2.412 | -0.252 | 0.049* |
|  | Static text-based encyclopedia | -0.637 | 0.147 | -4.329 | -0.449 | < .001*** |
| Dynamic text-based agent | Static text-based encyclopedia | -0.280 | 0.147 | -1.904 | -0.197 | 0.172 |

Note. * *p* < .05, ** *p* < .01, *** *p* < .001; p-values adjusted for comparing a family of 3

**Table S7. Results of post hoc comparisons for the univariate analyses of variance on cognitive and social-affective processes in Experiment 2**

| **Processing Fluency** |  | Mean Difference | *SE* | *t* | Cohen's *d* | *p*_bonf_ |
| --- | --- | --- | --- | --- | --- | --- |
| Dynamic text-based agent | Static text-based encyclopedia | -0.122 | 0.094 | -1.299 | -0.124 | 0.583 |
|  | Voice-based agent | 0.324 | 0.094 | 3.455 | 0.329 | 0.002** |
| Static text-based encyclopedia | Voice-based agent | 0.446 | 0.093 | 4.806 | 0.453 | < .001*** |
| **Social Presence** | | | | | | |
| Dynamic text-based agent | Static text-based encyclopedia | 0.964 | 0.167 | 5.776 | 0.550 | < .001*** |
|  | Voice-based agent | 0.272 | 0.167 | 1.625 | 0.155 | 0.314 |
| Static text-based encyclopedia | Voice-based agent | -0.693 | 0.165 | -4.186 | -0.395 | < .001*** |
| **Perceived Enjoyment** | | | | | | |
| Dynamic text-based agent | Static text-based encyclopedia | 0.452 | 0.144 | 3.131 | 0.298 | 0.005** |
|  | Voice-based agent | 0.663 | 0.145 | 4.583 | 0.437 | < .001*** |
| Static text-based encyclopedia | Voice-based agent | 0.211 | 0.143 | 1.475 | 0.139 | 0.422 |

Note. ** *p* < .01, *** *p* < .001; p-values adjusted for comparing a family of 3

**Table S8. Absolute cell sample sizes in Experiments 1 and 2**

|  | **Branding** | |
| --- | --- | --- |
|  | Unbranded | Branded |
|  | *n* | *n* |
| **Experiment 1** |  |  |
| **Presentation mode**  Voice-based agent  **Set**  A  B |  | 95  88 |
| Dynamic text-based agent  **Set**  A  B |  | 90  94 |
| Static text-based encyclopedia  **Set**  A  B |  | 96  93 |
| **Total** |  | **556** |
|  | Unbranded | Branded |
|  | *n* | *n* |
| **Experiment 2** |  |  |
| **Presentation mode**  Voice-based agent  **Set**  A  B | 58  55 | 54  57 |
| Dynamic text-based agent  **Set**  A  B | 54  53 | 54  55 |
| Static text-based encyclopedia  **Set**  A  B | 57  54 | 57  58 |
| **Total** | **666** | |

Note. Experiment 1 (top): Set A comprised low accuracy information on the topics Appendicitis, Bones, Wolf, and high accuracy information on Slovenia, Hookah, Titanic. Set B vice versa. Experiment 2 (bottom): Set A comprised low accuracy information on the topics Appendicitis and Hookah, and high accuracy information on Slovenia and Titanic. Set B vice versa.

**Table S9. Descriptive statistics, correlations, and alpha values for primary variables in Experiments 1 and 2**

| Variable | Experiment | *M* | *SD* | α | 1 | 2 | 3 | 4 |
| --- | --- | --- | --- | --- | --- | --- | --- | --- |
| 1. Information credibility | E1  E2 | 5.04  5.09 | 1.05  1.10 | .73  .67 | – |  |  |  |
| 1. Elaborate processing | E1  E2 | 85.95  75.17 | 16.52  22.15 | .83  .87 | .05  .09* | – |  |  |
| 1. Processing fluency | E1  E2 | 5.85  5.71 | 0.93  1.00 | .63  .68 | .17**  .23** | .08  .00 | – |  |
| 1. Social presence | E1  E2 | 3.20  3.11 | 1.88  1.80 | .96  .95 | .39**  .34** | .03  .10** | .09*  .03 | – |
| 1. Perceived enjoyment | E1  E2 | 4.94  4.61 | 1.44  1.54 | .95  .95 | .34**  .36** | .15**  .18** | .30**  .32** | .41**  .42** |

Note. The results of Experiment 1 (E1) are displayed in the first row (*N*_1_ = 556), the results of Experiment 2 (E2) in the second row (*N*_2_ = 666) of each variable. Cronbach’s α. * *p* < .05, ** *p* < .01 (both two-tailed).

**Table S10. Results of the univariate analyses of variance on social attraction in Experiment 1**

|  | Social Attraction | | |  |
| --- | --- | --- | --- | --- |
| Effect | *df* | *F* | *p* | η²_p_ |
| Presentation Mode | 1, 365 | 1.28 | .259 | .00 |
